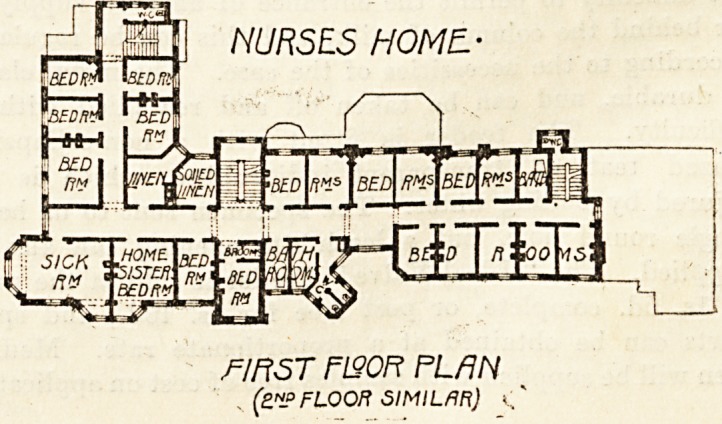# King's Norton Union Infirmary

**Published:** 1908-11-21

**Authors:** 


### HOSPITAL ADMINISTRATION.

### CONSTRUCTION AND ECONOMICS.

### KING'S NORTON UNION INFIRMARY.

The Infirmary, of which the buildings we illustrate to-day are an extension, was built in 1897 for the treatment of the sick poor of the large and populous district comprised within the King's Norton Union. The total accommodation for patients is now 486 beds, the new additions accounting for 228 out of that number.

The new wing for patients is planned on the usual lines of Poor-law infirmaries—*e.g.* the beds in the wards are arranged in couples instead of singly between the windows, and the wards are only 24 feet wide.

The ward pavilions are three storeys in height, and on each floor is a large ward for 24 beds with a smaller one for four beds opening out of it. This small ward is separated from the large ward by a glazed partition the full height of the ward, and is intended to be used either as a ward or as a day-room. Leading out of it is a covered-in balcony and a staircase for use as escape in case of fire. On the west side of the ward is a glazed verandah for consumptive patients.

The sanitary block is at the entrance end of the large ward, and is so planned that it can be approached directly from the large ward and also by a passage from the corridor, thus making it available for the two small wards at this part of the wing.

The corridor connecting the new wing with the existing infirmary is covered in on the ground floor, but not on the floors above. This corridor divides the new wing into two parts, each consisting of a large ward, two separation wards, duty-room, and bath-room; but in the case of the south portion the main ward is deferred for future extension. Of the two separation wards one contains four beds, the other two. The bath-rooms are fitted with two baths divided by a screen.

On the ground floor an operation-room with an anæsthetic-room adjoining is placed off the north side of the corridor. It would have been a great improvement if these rooms had been separated from the corridor by a lobby with double doors to cut off sound. A sterilising-room also suggests itself as desirable, if not necessary.

Another point that suggests itself is the inadequacy of the sink-rooms. A recess the size of a water-closet is not sufficient to contain the necessary sinks for washing vessels and for emptying bed-pans and urine-bottles and for scrubbing mackintoshes. The requirements of a Poor-law infirmary in this respect are not in any degree less than those of a general hospital; but in all buildings subject to the Local Government Board these points seem to be consistently ignored.

The Nurses' Home is a well-planned building and an excellent example of its kind. Accommodation is provided for 54 nurses in separate rooms with separate sitting-rooms for sisters and probationers, a reading-room, visitors' room, and a sick-room.

The isolation block has had added to it two wards for four adults or six children, with a nurse's room to each, together with a bath-room and dining-room for nurses.

The whole of these works have been planned and carried out under the superintendence of Messrs. C. Whitwell and Son, of Birmingham, at a total cost—including lifts, electric light, and furnishing—of about £47,000.

## Figures and Tables

**Figure f1:**
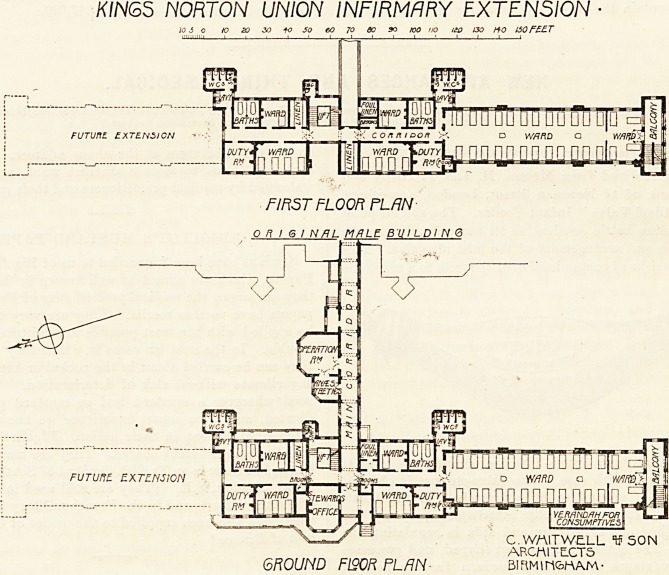


**Figure f2:**
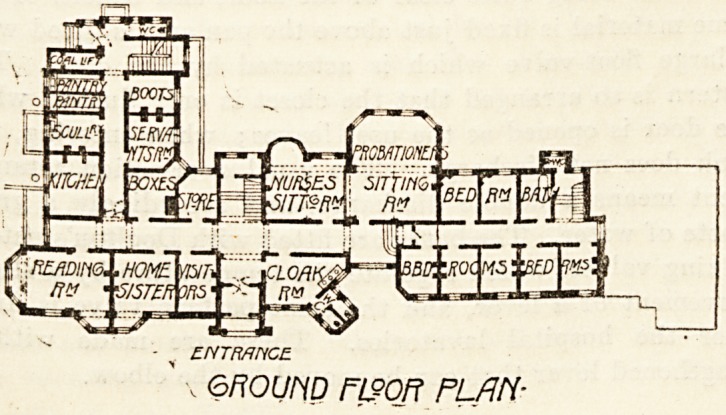


**Figure f3:**